# Causal relationship between sarcopenia and osteoarthritis: a bi-directional two-sample mendelian randomized study

**DOI:** 10.1186/s40001-023-01322-0

**Published:** 2023-09-09

**Authors:** Jiyong Yang, Peng Liu, Shuai Wang, Tao Jiang, Yilong Zhang, Wengang Liu

**Affiliations:** 1 The Fifth Clinical College of Guangzhou, University of Chinese Medicine, Guangzhou, China; 2 The First Affiliated Hospital of Sun Yat-sen University, Guangzhou, China; 3 Department of Orthopedics, Guangdong Second Traditional Chinese Medicine Hospital, Guangzhou, China

**Keywords:** Osteoarthritis, Sarcopenia, Sarcopenic osteoarthritis, Mendelian randomization, Causal relationship

## Abstract

**Background:**

Previous studies have shown that osteoarthritis (OA) and sarcopenia (SP) are closely related to each other, but the causal relationships between them have not been established. The aim of this study was to investigate the causal associations between OA and SP via a bi-directional Mendelian randomization (MR) approach.

**Methods:**

A bi-directional two-sample MR was adopted to research the causal relationship between SP and OA. The instrumental variables for SP and four types of OA: KOA, HOA, total knee replacement (TKR) and total hip replacement (THR) were derived from published large genome-wide association studies (GWAS). The inverse variance weighted (IVW), MR-Egger and weighted median estimator (WME) methods were used to estimate bi-directional causal effects.

**Results:**

Low grip strength (GS) did not have a causal effect on four types of OA (KOA: OR = 1.205, 95% CI 0.837–1.734, *p* = 0.316; HOA: OR = 1.090, 95% CI 0.924–1.609, *p* = 0.307; TKR: OR = 1.190, 95% CI 1.084–1.307, *p* = 0.058; THR: OR = 1.035, 95% CI 0.792–1.353, *p* = 0.798), while appendicular lean mass (ALM) had a causal effect on four types of OA (KOA: OR = 1.104, 95% CI 1.041–1.171, *p* = 0.001; HOA: OR = 1.151, 95% CI 1.071–1.237, *p* < 0.001; TKR: OR = 1.114, 95% CI 1.007–1.232, *p* < 0.001; THR: OR = 1.203, 95% CI 1.099–1.316, *p* < 0.001). In the reverse direction, KOA or HOA did not have a significant causal effect on both GS and ALM (KOA-GS: OR = 1.077, 95% CI 0.886–1.309, *p* = 0.458; KOA-ALM: Beta = 0.004, *p* = 0.892; HOA-GS: OR = 1.038, 95% CI 0.981–1.099, *p* = 0.209; HOA-ALM: Beta = − 0.017, *p* = 0.196; TKR-GS: OR = 0.999, 95% CI 0.739–1.351, *p* = 0.997; TKR-ALM: Beta = 0.018, *p* = 0.501; THR-GS: OR = 1.037, 95% CI 0.978–1.101, *p* = 0.222; THR-ALM: Beta = − 0.023,* p* = 0.081).

**Conclusions:**

The present study suggests that SP may have a causal effect on OA through changes in muscle composition rather than muscle strength, while little evidence was provided for the causal effect of OA on SP.

**Supplementary Information:**

The online version contains supplementary material available at 10.1186/s40001-023-01322-0.

## Introduction

With a worldwide population aging, chronic musculoskeletal disorders have imposed a tremendous burden on society [1]. Among them, osteoarthritis (OA) and sarcopenia (SP) are two common diseases. OA is characterized by the destruction and loss of articular cartilage as its main pathological feature, but all joint tissues and even extra-articular structures are involved in some form [2, 3]. The two most common types in clinical practice are knee osteoarthritis (KOA) and hip osteoarthritis (HOA). Sarcopenia was originally defined in 1989 as the age-related loss of muscle mass, but gradually expanded to include muscle strength, muscle mass and physical performance [4, 5]. As two age-related diseases, the prevalence of OA and SP is annually increasing [6, 7]. Unfortunately, at present, we do not have an effective treatment for these two diseases [8, 9]. Moreover, the coexistence of these two conditions: sarcopenic OA, which is frequently seen in clinical practice, could exacerbate the risk of falls and compromise the quality of life [10, 11].

Preliminary clinical studies have shown a strong correlation between OA and SP and suggested that one condition can increase the possibility of developing the other, especially in the OA of the lower limbs [12–14]. Initially, OA and SP are interconnected by biomechanical factors represented by muscle strength [15–17]. With the in-depth study of bone-muscle crosstalk, the researchers found that the relationship between OA and SP can also be mediated biologically [18–20]. The balance between lean mass and fat mass in muscle would lead to the dysregulation of multiple myokines. These myokines consequently affect cartilage gene expression in terms of formation and homeostasis [21–23]. On the other hand, several molecules released by bone structures can also modulate muscles, such as Indian hedgehog and undercarboxylated osteocalcin [24, 25]. However, none of the previous studies demonstrated the causality relationship between OA and SP. Evaluating the causality between OA and SP may provide new strategies for prevention, diagnosis, and treatment of OA, SP and sarcopenic OA.

Recent studies have extensively applied the Mendelian randomization (MR) approach to provide evidence for the causal relationships between exposures and outcomes [26–28]. The identification of single nucleotide polymorphisms (SNPs) associated with common complex diseases has been greatly facilitated by genome-wide association studies (GWAS) [29]. By applying SNPs as instrumental variables (IVs), the MR method can obtain a robust causal estimate independent of postnatal lifestyle or environmental factors [30]. Several studies have identified causal factors leading to OA or SP [26–28], but no MR study has examined causal relationships between OA and SP. Therefore, the aim of our study was to investigate the causal associations between OA of lower limbs and SP via a bi-directional MR approach. We assumed that SP and OA have a significant casual effect on each other.

## Materials and methods

### Study overview

**Fig. 1:**
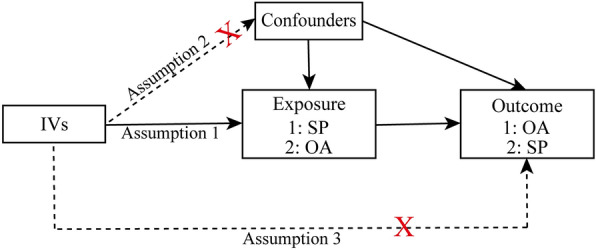
Schematic representation of the three assumptions of Mendelian randomization and current study design. Assumption 1: the IVs should be closely associated with exposures; Assumption 2: the IVs selected are not associated with potential confounders; Assumption 3: the IVs should affect results dependently through exposure, but not the direct correlation. This bi-directional MR analysis was performed in two steps: sarcopenia was studied as exposure while osteoarthritis was studied as outcome in the first step, whereas the second step was reversed. *IVs* instrumental variable, *SP* sarcopenia, *OA* osteoarthritis

This study is based on the existing datasets of large sample genome-wide association studies. Through the two-sample MR, which was performed and reported according to the Strengthening the reporting of observational studies in epidemiology using Mendelian randomization (STROBE-MR) guidelines [31], the causal relationship between sarcopenia-related traits and OA were explored using genetic variants for exposure as instrument variables (IV). In the first step of this analysis, SP-related traits were investigated as exposure, while OA and its subtypes were investigated as outcome. The second step reversed the exposure and outcome analyses (Fig. 1). To minimize bias, all participants of the selected datasets were restricted to European ancestry. Meanwhile, this analysis was conducted using data from approved studies that received informed consent from all participants.

### Data sources

In this study, a total of four phenotypes of OA in the lower limbs were investigated: KOA, HOA, total knee replacement (TKR), and total hip replacement (THR). Summary-level data of OA were extracted from the largest genome-wide meta-analysis to date across 826,690 individuals which include the 11 OA phenotypes [32]. In the current study, the data from KOA (n = 62,497), HOA (n = 36,445), TKR (n = 18,200), THR (n = 23,021), and a max of healthy controls (n = 333,557) were used. Based on the data available in the cohort, OA was defined as self-reported, clinically diagnosed, ICD10 codes, or radiographic. OA-free or population-based controls were included or excluded based on ICD codes.

Appendicular lean mass (ALM) and grip strength (GS) are potentially important as measure of muscle mass and quality in elderly people which were selected as the sarcopenia-related traits in this study [5]. The summary‐level statistics of ALM were obtained from a genome-wide association analysis which included 450,243 UK Biobank participants [33]. ALM was measured using bioelectrical impedance analysis by Tanita BC 418ma Body Fat Analyzer, and its measurement accuracy was validated using the DEXA method. The low GS data were achieved from a previous muscle weakness-related study, which comprised 256,523 individuals of European descent aged 60 years or older [34]. GS was measured using a Jamar J00105 hydraulic hand dynamometer, and the maximum hand grip strength was recorded in whole kilogram force units, and the low GS was defined as grip strength < 30 kg for males and < 20 kg for females. The original publications provide more details about phenotypes, genotype quality control, and related association analyses.

### Selection and validation of genetic instrumental variables

For a stable MR analysis, the selections of IVs are vital which should be strictly adhered to three assumptions: (1) the chosen IVs are robustly related to the investigation exposure; (2) no confounding variables exist between the exposure and outcome affecting the chosen IVs; (3) other than by their association with the exposure, the chosen IVs have no impact on the outcome. To satisfy these assumptions, we chose independent SNPs without linkage disequilibrium (clumping *r*^2^ = 0.001 and kb = 10,000) and associated with the exposure at the genome‐wide significance level (*p* < 5 × 10^–8^) as instrumental SNPs. Furthermore, the *F* statistic were calculated to evaluate the strength of selected IVs and an *F* < 10 was considered dubious bias which was removed in the MR analysis. *F* statistic was calculated via the following formula: *F* = [*R*^2^/(1-*R*^2^)] × [(*N*-*K*-1)/*K*], where *N* = GWAS sample size, *K* = number variants comprising the instrument. R^2^ was calculated using the following formula: *R*^2^ = [beta.exposure^2^]/[se.exposure^2^ × *N* + beta.exposure^2^], where beta.exposure = SNP exposure effect and se. exposure = standard error of SNP exposure effect [31]. Finally, we detected the associations of all SNPs with both outcome and confounders (For the SP-related traits, nutritional intake, lifestyle variables, age, sex, and chronic wasting disease are considered as confounders. While age, sex, and body mass index are potential risk factors of OA) in the Phenoscanner database (http://www.phenoscanner.medschl.cam.ac.uk/) and deleted those with *p* < 5 × 10^−8^.

### Statistical analysis

We adopt the inverse variance weighted (IVW) method as the primary statistical approach to evaluate the bi-directional relation between SP and OA, which was considered to be the most robust indicator in the absence of evidence of directional pleiotropy among the selected IVs (*p* > 0.05 for MR-Egger cutoff) [35]. To estimate whether there was a high heterogeneity among the IVs selected for analysis, Cochran’s* Q* test was conducted. In the absence of significant heterogeneity, the fixed-effects model would be adopted, otherwise, a random-effects model was conducted. As complements to IVW method, MR-Egger and the weighted median estimator (WM) were also used to estimate causal effects between SP-related traits and OA. The MR-Egger method allows all SNPs with horizontal pleiotropic effects to be unbalanced or directed, while the WM method can obtain a robust causal association evaluation even with the presence of up to 50% of invalid IVs [36]. For further validation of the MR causal effect estimation, we also used the MR pleiotropy residual sum and outlier (MR-PRESSO) method to detect pleiotropic effects in the sensitivity analysis which can identify and remove possible pleiotropic IVs. After remove the outliers, the IVW method was repeated [37]. In this bi-directional two-sample MR analysis, R software was used to conduct all analysis using TwoSampleMR and MR-PRESSO packages. Due to multiple testing, we adopted the Bonferroni method to adjust the significance level in the presence of multiple tests, utilizing a stricter *p*-value threshold of 0.05/2 × 4 = 0.00625. The analytical results with a *p*-value between 0.0125 and 0.05 are considered nominally significant results.

## Results

### Influence of sarcopenia-related traits on OA

For the GS, we initially identified 17 IVs which all had a *F* statistic > 10. After integrating the GWAS data of KOA, HOA, TKR, and THR and excluding SNPs associated with outcomes or confounding factors, we selected 8, 7, 6, and 7 LD-independent and appropriate instrumental variables for MR analysis of GS-KOA, HOA, TKR, and THR (Additional file 1: Tables S1–S4). According to the heterogeneity test, the random-effects model was used in the primary MR analysis of GS-KOA, TKR, and THR, and the fixed-effects model was used in the analysis of GS-HOA (Table 1). The primary IVW analyses provide no evidence for the causal relationship between the GS and four types of OA (KOA: OR = 1.205, 95% CI 0.837–1.734, *p* = 0.316; HOA: OR = 1.090, 95% CI 0.924–1.609, *p* = 0.307; TKR: OR = 1.659, 95% CI 0.983–2.803, *p* = 0.058; THR: OR = 1.035, 95% CI 0.792–1.353, *p* = 0.798). The MR pleiotropy test revealed no horizontal pleiotropy in the analysis of four types of OA. 3 outlier IVs were identified in the MR‐PRESSO analysis of GS-KOA. When the outliers were removed, the IVW results still not found a significant causal association between GS and KOA. Overall, the MR analyses supported the notion that GS was not causally associated with OA.

**Table 1:** MR estimates from different methods of assessing the causal effect of low grip strength on osteoarthritis

Exposures	Outcomes	No. of IVs	Heterogeneity test	MR-egger	MR results	OR (95% CI)	*p*
			Cochran’s q (*p*)	Intercept (*p*)	Method		
GS	KOA	8	65.242 (< 0.001)	0.009 (0.784)	IVW	1.205 (0.837, 1.734)	0.316
					MR-egger	1.093 (0.840,1.421)	0.529
					WM	1.088 (0.883,1.340)	0.431
					PRESSO (3)	1.122 (0.927, 1.358)	0.304
GS	HOA	7	5.395 (0.494)	0.012 (0.540)	IVW	1.090 (0.924,1.609)	0.307
					MR-egger	0.862 (0.419,1.771)	0.703
					WM	1.079 (0.924,1.287)	0.501
					PRESSO (0)	1.090 (0.924,1.609)	0.307
GS	TKR	6	23.799 (< 0.001)	0.016 (0.767)	IVW	1.659 (0.983,2.803)	0.058
					MR-egger	1.601 (0.929,2.755)	0.774
					WM	1.582 (1.102,2.272)	0.013
					PRESSO (0)	1.659 (0.983,2.803)	0.058
GS	THR	7	10.208 (< 0.001)	0.003 (0.909)	IVW	1.035 (0.792,1.353)	0.798
					MR-egger	1.181 (0.771,1.808)	0.003
					WM	1.148 (0.849,1.553)	0.369
					PRESSO (0)	1.035 (0.792,1.353)	0.798

*MR* Mendelian randomization, *IVs* instrumental variables, *GS* grip strength, *KOA* knee osteoarthritis, *HOA*, hip osteoarthritis, *TKR* total knee replacement, *THR* total hip replacement, *ALM* appendicular lean mass, *OR* odds ratio, *CI* confidence interval, *IVW* inverse variance weighted, *WM* weighted median, *PRESSO* Pleiotropy RESidual Sum and Outlier

For the ALM, we initially identified 690 SNPs which all had a *F* statistic > 10. Following harmonization with GWAS data for KOA, HOA, TKR, and THR and exclusion of SNPs associated with outcomes or confounders, 486, 487, 487, and 486 LD-independent and appropriate IVs were selected for MR analysis of GS-KOA, HOA, TKR, and THR (Additional file 1: Tables S5 to S8). The random-effects model was employed in the primary MR analysis of four types of OA due to the presence of heterogeneity (Table 2). The primary IVW analyses indicated that ALM had a significant causal effect on four types of OA (KOA: OR = 1.104, 95% CI 1.041–1.171, *p* = 0.001; HOA: OR = 1.151, 95% CI 1.071–1.237, *p* < 0.001; TKR: OR = 1.114, 95% CI 1.007–1.232, *p* = 0.036; THR: OR = 1.203, 95% CI 1.099–1.316, *p* < 0.001). Despite the detection of horizontal pleiotropy in the analysis of ALM-TKR and THR by the MR pleiotropy test, as well as several potential pleiotropic IVs for all four types of OA identified by MR-PRESSO, removal of outliers did not alter the significant causal association between ALM and THR revealed by IVW results. In the analysis of TKR, the removal of outliers made the difference more significant (OR = 1.114, 95% CI 1.007–1.232, *p* < 0.001). Based on all of the MR analyses, ALM had a significant causal effect on OA.

**Table 2:** MR estimates from different methods of assessing the causal effect of appendicular lean mass on osteoarthritis

Exposures	Outcomes	No. of IVs	Heterogeneity test	MR-egger	MR results	OR (95% CI)	*p*
			Cochran’s q (*p*)	Intercept (*p*)	Method		
ALM	KOA	486	993.209 (< 0.001)	0.003 (0.079)	IVW	1.104 (1.041,1.171)	0.001
					MR-egger	0.967 (0.825,1.134)	0.679
					WM	1.0.85 (1.009,1.166)	0.037
					PRESSO (9)	1.134 (1.073, 1.199)	< 0.001
ALM	HOA	487	890.952 (< 0.001)	0.004 (0.052)	IVW	1.151 (1.071,1.237)	< 0.001
					MR-egger	0.961 (0.791,1.168)	0.690
					WM	1.154 (1.056,1.261)	0.002
					PRESSO (7)	1.155 (1.081,1.235)	< 0.001
ALM	TKR	487	917.707 (< 0.001)	0.005 (0.034)	IVW	1.114 (1.007,1.232)	0.036
					MR-egger	0.845 (0.643,1.112)	0.321
					WM	1.114 (1.007,1.232)	0.098
					PRESSO (11)	1.190 (1.084,1.307)	< 0.001
ALM	THR	486	894.639 (< 0.001)	0.006 (0.014)	IVW	1.203 (1.099,1.316)	< 0.001
					MR-egger	0.906 (0.711,1.156)	0.429
					WM	1.143 (1.020,1.281)	0.021
					PRESSO (8)	1.226 (1.129,1.332)	< 0.001

*MR* Mendelian randomization, *IVs* instrumental variables, *GS* grip strength, *KOA* knee osteoarthritis, *HOA* hip osteoarthritis, *TKR* total knee replacement, *THR* total hip replacement, *ALM* appendicular lean mass, *OR* odds ratio, *CI* confidence interval, *IVW* inverse variance weighted, *WM* weighted median, *PRESSO* Pleiotropy RESidual Sum and Outlier

### Influence of OA on sarcopenia-related traits

For the KOA, we initially identified 23 SNPs which all had a *F* statistic > 10. Following harmonization with GWAS data for GS and ALM, and exclusion of SNPs associated with outcomes or confounders, 15 and 13 LD-independent and appropriate IVs were selected for MR analysis of KOA-GS and ALM, respectively (Additional file 1: Tables S9 to S10). As indicated by the heterogeneity test, the random-effects model was adopted both in the primary MR analysis of KOA-GS and KOA-ALM (Table 3). The primary IVW analyses do not provide any evidence supporting a causal relationship between KOA and GS or ALM (KOA-GS: OR = 1.077, 95% CI 0.886–1.309, *p* = 0.458; KOA-ALM: Beta = 0.004, *p* = 0.892). The MR pleiotropy test revealed no horizontal pleiotropy. Although 3 outliers were identified in the MR‐PRESSO analysis of KOA-ALM, the IVW results were consistent with previous. Overall, the MR analyses supported the notion that KOA was not causally associated with SP-related traits.

**Table 3:** MR estimates from different methods of assessing the causal effect of knee and hip osteoarthritis on sarcopenia

Exposures	Outcomes	No. of IVs	Heterogeneity test	MR-egger	MR results	Beta/OR (95% CI)	*p*
			Cochran’s q (*p*)	Intercept (*p*)	Method		
KOA	GS	15	77.246 (< 0.001)	− 0.023 (0.491)	IVW	1.077 (0.886,1.309)	0.458
					MR-egger	1.104 (0.956,1.275)	0.429
					WM	1.175 (1.026, 1.349)	0.019
					PRESSO (0)	1.077 (0.886,1.309)	0.458
KOA	ALM	13	59.001 (< 0.001)	0.003 (0.799)	IVW	0.004	0.892
					MR-egger	0.058	0.789
					WM	− 0.027	0.168
					PRESSO (3)	0.019	0.107
HOA	GS	22	59.120 (< 0.001)	− 0.009 (0.380)	IVW	1.106 (1.023,1.196)	0.012
					MR-egger	1.231 (0.962,1.557)	0.113
					WM	1.035 (0.961,1.113)	0.365
					PRESSO (4)	1.038 (0.981,1.099)	0.209
HOA	ALM	13	76.162 (< 0.001)	0.002(0.713)	IVW	− 0.015	0.451
					MR-egger	− 0.042	0.579
					WM	− 0.004	0.772
					PRESSO (4)	− 0.017	0.196

*MR* Mendelian randomization, *IVs* instrumental variables, *GS* grip strength, *KOA* knee osteoarthritis, *HOA* hip osteoarthritis, *ALM* appendicular lean mass, *OR* odds ratio, *CI* confidence interval, *IVW* inverse variance weighted, *WM* weighted median, *PRESSO* Pleiotropy RESidual Sum and Outlier

For the HOA, we initially identified 29 IVs which all had a *F* statistic > 10. Following harmonization with GWAS data for GS and ALM, and exclusion of SNPs associated with outcomes or confounders, 22 and 13 LD-independent and appropriate IVs were selected for MR analysis of HOA-GS and ALM, respectively (Additional file 1: Tables S11 to S12). As indicated by the heterogeneity test, the random-effects model was adopted both in the primary MR analysis of HOA-GS and HOA-ALM, and the MR pleiotropy test revealed no horizontal pleiotropy. Although the primary IVW analyses revealed a significant causal relationship between HOA and GS (OR = 1.106, 95% CI 1.023–1.196, *p* = 0.012), the IVW results did not find a significant causal association between HOA and GS (OR = 1.038, 95% CI 0.981–1.099, *p* = 0.209) after removing outlier IVs identified in the MR-PRESSO analysis. In the analysis of HOA-ALM, the primary IVW analyses and removal of outliers both provided no evidence for the causal relationship between the HOA and ALM (IVW: Beta = − 0.025, *p* = 0.451; PRESSO: Beta = − 0.017, *p* = 0.196), which was also consistent with the results of other methods. In total, there was no significant causal relationship between HOA and SP-related traits.

For the TKR, we initially identified 9 IVs which all had a *F* statistic > 10. Following harmonization with GWAS data for GS and ALM, and exclusion of SNPs associated with outcomes or confounders, 5 LD-independent and appropriate IVs were both selected for MR analysis of HOA-GS and ALM (Additional file 1: Tables S13 to S14). As indicated by the heterogeneity test, the random-effects model was adopted both in the primary MR analysis of TKR-GS and ALM, and the MR pleiotropy test revealed no horizontal pleiotropy (Table 4). In the analysis of TKR-GS and ALM, the primary IVW analyses and other methods provided no evidence for the causal relationship between the TKR and GS or ALM (TKR-GS: OR = 0.999, 95% CI 0.739–1.351, *p* = 0.997; TKR-ALM: Beta = 0.018, *p* = 0.501). No outlier was identified in the MR‐PRESSO analysis. The overall findings indicated the absence of a statistically significant causal association between TKR and SP-related traits.

**Table 4:** MR estimates from different methods of assessing the causal effect of total knee and hip replacement on sarcopenia

Exposures	Outcomes	No. of IVs	Heterogeneity test	MR-egger	MR results	Beta/OR (95% CI)	*p*
			Cochran’s q (*p*)	Intercept (*p*)	Method		
TKR	GS	5	56.813(< 0.001)	0.047(0.758)	IVW	0.999(0.739,1.351)	0.997
					MR-egger	1.017(0.851,1.215)	0.864
					WM	1.138(0.968, 1.340)	0.019
					PRESSO (0)	0.999(0.739,1.351)	0.997
TKR	ALM	5	27.341(< 0.001)	0.005(0.836)	IVW	0.018	0.501
					MR-egger	0.043	0.886
					WM	0.005	0.733
					PRESSO (0)	0.018	0.501
THR	GS	22	59.120(< 0.001)	− 0.004(0.749)	IVW	1.037(0.978,1.101)	0.222
					MR-egger	1.074(0.864,1.336)	0.528
					WM	1.012(0.961,1.083)	0.519
					PRESSO (0)	1.037(0.978,1.101)	0.222
THR	ALM	17	83.332 (< 0.001)	0.007(0.157)	IVW	− 0.023	0.081
					MR-egger	− 0.087	0.072
					WM	− 0.023	0.235
					PRESSO (4)	− 0.012	0.158

*MR* Mendelian randomization, *IVs* instrumental variables, *GS* grip strength, *KOA* knee osteoarthritis, *HOA* hip osteoarthritis, *TKR* total knee replacement, *THR* total hip replacement, *ALM* appendicular lean mass, *OR* odds ratio, *CI* confidence interval, *IVW* inverse variance weighted, *WM* weighted median, *PRESSO* Pleiotropy RESidual Sum and Outlier

For the THR, we initially identified 34 IVs which all had a *F* statistic > 10. Following harmonization with GWAS data for GS and ALM, and exclusion of SNPs associated with outcomes or confounders, 22 and 17 LD-independent and appropriate IVs were both selected for MR analysis of THR-GS and ALM, respectively (Additional file 1: Tables S15 to S16). As indicated by the heterogeneity test, the random-effects model was adopted both in the primary MR analysis of THR-GS and ALM, and the MR pleiotropy test revealed no horizontal pleiotropy (Table 4). In the analysis of THR-GS and ALM, the primary IVW analyses and other methods provided no evidence for the causal relationship between the THR and GS or ALM (THR-GS: OR = 1.037, 95% CI 0.978–1.101, *p* = 0.222; THR-ALM: Beta = − 0.023,* p* = 0.081). Although 4 outliers were identified in the MR‐PRESSO analysis of THR-ALM, the IVW results were consistent with previous. The overall findings indicated the absence of a statistically significant causal association between THR and SP-related traits.

## Discussion

This study aimed to explore the causal relationships between SP and OA via a bi-directional two-sample MR approach. To the best of our knowledge, our MR study is the first to evaluate the bi-directional causal link between SP and OA comprehensively. Based on our results, there is a causal effect of SP on OA, while conversely, we did not observe a significant causal effect of OA on SP. Moreover, our findings indicated a causal relationship between SP and OA through the mediation of ALM.

Previous clinical studies have demonstrated the positive correlation between SP and OA. In a cross-sectional study, Suh et al. found that high fat mass and low lower extremity muscle mass were correlated with the presence and intensity of KOA [17]. The similar results were also found in the HOA, which were featured by the proportionally higher fat mass, and lower lean body mass [38, 39]. Furthermore, with better awareness of the importance of SP on OA, multiple long follow-up, large sample cohort studies were conducted to observe the relationship between them. In a cohort of healthy older population with no clinically diagnosed, symptomatic KOA and knee pain, ALM and GS was associated with the development of KOA and knee pain 5 years later, respectively [40]. In another large longitudinal cohort study, the body composition of fat and muscle mass was also associated with KOA risk within a 60-month follow-up period [41]. Meanwhile, compared to the isolated OA, the quality of life was more compromised and the likelihood of patients being referred for surgery was higher in the presence of SP [14, 42]. However, there was still a lack of high-level evidence-based evidence for the causality of SP for OA, for instance, randomized controlled studies. Our MR study found a significant causal effect of SP on OA, which provided additional evidence to support causality between them.

A diagnosis of SP can be confirmed by the presence of both low muscle strength and low muscle quantity or quality, as one alone is not sufficient [43]. Consistent with previous observational studies mentioned above, our study showed that four types of OA were causally associated with a lower ALM. However, OA is not causally affected by low GS, unlike what is expected. This may be due to several reasons. First, in the summary-level data of GS, the low GS was defined as grip strength < 30 kg for males and < 20 kg for females, which was recommended in 2010 by The European Working Group on Sarcopenia in Older People (EWGSOP) [44]. However, EWGSOP2 updated the cutoff values of low GS in 2018: < 27 kg for males and < 16 kg for females [43]. While in the Foundation for the National Institutes of Health Sarcopenia Project, the cutoff values of low GS are < 26 kg for males and < 16 kg for females [45]. The assessment of the causal effect of low GS on OA may have been influenced by the differences in the criteria for low GS in SP. Furthermore, although higher muscle strength can better maintain joint stability to protect the joint, some clinical studies also indicated that greater muscle strength is not always protective. Chaisson et al. reported that men and women with higher GS were both associated with a greater risk of developing incident radiographic OA at hand [46]. Similar results were also reported by Sharma et al. in KOA, in which they found greater quadriceps strength was associated with increased likelihood of OA progression in malaligned knees and lax knees [47]. Further research is required to determine the impact of muscle strength on OA.

On the other hand, muscle composition changes are believed to be the primary contributor to OA in SP. Accumulating evidence suggested muscle as a paracrine and endocrine organ which can secrete a variety of myokines to modulate the bone, including irisin, insulin-like growth factor-1, myostatin, and interleukin 6 [48]. The dysregulation of these myokines were proven to be relevant in the development and progress of OA [49, 50]. Simultaneously, the increased fat mass (decreased lean mass) is another important factor for explaining the potential causal association between SP and OA [51]. Fat tissue can secrete a variety of deleterious cytokines for both cartilage and muscle, including inflammatory cytokines, leptin, and adiponectin. The cumulative release of these cytokines eventually leads to systemic low-grade inflammation, which has also been considered the basis of OA [23, 52]. Our study results suggest that ALM has a significant causal effect on OA. Similar findings were reported by Liu et al. who found a positive causal relationship between ALM and bone mineral density. However, there was no evidence of a causal association between low GS and bone mineral density [53]. These findings suggest that changes in muscle composition may play a crucial role in the muscle–bone crosstalk mechanism.

In the reverse direction, although we found a nominally significant result for the relationship between HOA and GS, the rest results did not support the causal effect of OA on SP. Compared to studies on the influence of SP on OA, fewer studies have focused on the impact of OA on SP. Most recently, Francesco et al. conducted a systematic review and meta-analysis of the prevalence of SP in KOA [54]. It is noteworthy, though, that they only included four cross-sectional studies in their analysis, even though their results indicated a greater prevalence of SP in patients with KOA than in non-KOA patients. Besides, from the perspective of bone-muscle crosstalk, there was limited research regarding the possible direct influence of chondrocytes on muscular cells in vitro and in animal research. Some researchers argued that atrophy of muscles in osteoarthritis would be caused more by the functional impairment caused by pain than by direct biomolecular factors inhibiting muscle development [50]. Therefore, well-designed epidemiological, molecular mechanism and MR experiments are required to determine the causality of OA on SP in the future.

Previously, studies have tried to analyze the causal relationship between SP and OA, but no MR analysis has been conducted to investigate bi-directional causal link between SP and OA. As a result of the MR approach, we were able to avoid most confounding factors and reverse causality associated with traditional observational studies, which always bias these studies. In the present study, we selected IVs based strictly on the three hypotheses of the MR study, which makes our results more reliable. Furthermore, we adopted various methods as well as sensitivity tests to assess the causal relationship between SP and OA, which can generate more robust results. Nevertheless, there were some limitations in our study. First, the results from other MR methods, including MR-Egger and weighted median did not fully align with the IVW method in the MR analysis. However, based on the principle of method selection, IVW estimated results can be preferred if there is no pleiotropy present. Second, only summary statistics were collected, so it was not possible to assess the effect of age or gender separately. Moreover, the selected datasets were restricted to European ancestry, it is unknown if similar results would be obtained from other ancestries. Lastly, although confounding has been addressed in this study, the potential impact of third-party factors cannot be ruled out. Therefore, the results may not follow a linear pattern and require additional verification.

## Conclusion

In conclusion, the results of our MR analysis supported a causal association between SP and OA risk, while little evidence was provided for the causal effect of OA on SP. Our findings emphasize the importance of muscle mass in this causal relationship. This should be considered and validated in future OA studies to develop prevention or treatment strategies. More in-depth studies are needed in the future to demonstrate the causal effect of OA on SP.

### Supplementary Information

**Additional file 1:** **Table S1.** The selected SNPs in the analysis of GS-KOA; **Table S2.** The selected SNPs in the analysis of GS-HOA; **Table S3.** The selected SNPs in the analysis of GS-TKR; **Table S4.** The selected SNPs in the analysis of GS-THR; **Table S5.** The selected SNPs in the analysis of ALM-KOA; **Table S6.** The selected SNPs in the analysis of ALM-HOA; **Table S7.** The selected SNPs in the analysis of ALM-TKR; **Table S8.** The selected SNPs in the analysis of ALM-THR; **Table S9.** The selected SNPs in the analysis of KOA-GS; **Table S10.** The selected SNPs in the analysis of KOA-ALM; **Table S11.** The selected SNPs in the analysis of HOA-GS; **Table S12. **The selected SNPs in the analysis of HOA-ALM. **Table S13.** The selected SNPs in the analysis of TKR-GS; **Table S14.** The selected SNPs in the analysis of TKR-ALM. **Table S15.** The selected SNPs in the analysis of THR-GS; **Table S16.** The selected SNPs in the analysis of THR-ALM.

## Data Availability

All the data used in this study are available at GWAS datasets.

## References

[1] Liu S, Wang B, Fan S, Wang Y, Zhan Y, Ye D (2022). Global burden of musculoskeletal disorders and attributable factors in 204 countries and territories: a secondary analysis of the global burden of disease 2019 study. BMJ Open.

[2] Motta F, Barone E, Sica A, Selmi C (2022). Inflammaging and osteoarthritis. Clin Rev Allerg Immu.

[3] Coaccioli S, Sarzi-Puttini P, Zis P, Rinonapoli G, Varrassi G (2022). Osteoarthritis: new insight on its pathophysiology. J Clin Med.

[4] Rosenberg IH (1997). Sarcopenia: origins and clinical relevance. J Nutr.

[5] Cruz-Jentoft AJ, Bahat G, Bauer J, Boirie Y, Bruyère O, Cederholm T (2019). Sarcopenia: revised European consensus on definition and diagnosis. Age Ageing.

[6] Keevil VL, Romero-Ortuno R (2015). Ageing well: a review of sarcopenia and frailty. P Nutr Soc.

[7] Prieto-Alhambra D, Judge A, Javaid MK, Cooper C, Diez-Perez A, Arden NK (2014). Incidence and risk factors for clinically diagnosed knee, hip and hand osteoarthritis: influences of age, gender and osteoarthritis affecting other joints. Ann Rheum Dis.

[8] Feike Y, Zhijie L, Wei C (2021). Advances in research on pharmacotherapy of sarcopenia. Aging Med.

[9] Abramoff B, Caldera FE (2019). Osteoarthritis pathology, diagnosis, and treatment options. Med Clin N Am.

[10] Chung SM, Hyun MH, Lee E, Seo HS (2016). Novel effects of sarcopenic osteoarthritis on metabolic syndrome, insulin resistance, osteoporosis, and bone fracture: the national survey. Osteoporosis Int.

[11] Iijima H, Aoyama T (2021). Increased recurrent falls experience in older adults with coexisting of sarcopenia and knee osteoarthritis: a cross-sectional study. BMC Geriatr.

[12] Kim H-J, Hong Y-H (2022). Age-related low skeletal muscle mass correlates with joint space narrowing in knee osteoarthritis in a South Korean population: a cross-sectional, case-control study. Yeungnam Univ J Med.

[13] Gao Q, Hu K, Yan C, Zhao B, Mei F, Chen F (2021). associated factors of sarcopenia in community-dwelling older adults: a systematic review and meta-analysis. Nutrients.

[14] Jeanmaire C, Mazières B, Verrouil E, Bernard L, Guillemin F, Rat A-C (2018). Body composition and clinical symptoms in patients with hip or knee osteoarthritis: results from the KHOALA cohort. Semin Arthritis Rheu.

[15] Ruhdorfer A, Wirth W, Eckstein F (2017). Association of knee pain with a reduction in thigh muscle strength—a cross-sectional analysis including 4553 osteoarthritis initiative participants. Osteoarthr Cartil.

[16] Yamauchi K, Suzuki S, Kato C, Kato T (2020). Atrophy of individual thigh muscles measured by MRI in older adults with knee osteoarthritis: a cross-sectional study. Ann Phys Rehabilit Med.

[17] Suh DH, Han KD, Hong JY, Park JH, Bae JH, Moon YW (2016). Body composition is more closely related to the development of knee osteoarthritis in women than men: a cross-sectional study using the fifth Korea national health and nutrition examination survey (KNHANES V-1, 2). Osteoarthr Cartil.

[18] He C, He W, Hou J, Chen K, Huang M, Yang M (2020). Bone and muscle crosstalk in aging. Front Cell Dev Biol.

[19] Li G, Zhang L, Wang D, AIQudsy L, Jiang JX, Xu H (2019). Muscle-bone crosstalk and potential therapies for sarco-osteoporosis. J Cell Biochem.

[20] Scimeca M, Piccirilli E, Mastrangeli F, Rao C, Feola M, Orlandi A (2017). Bone morphogenetic proteins and myostatin pathways: key mediator of human sarcopenia. J Transl Med.

[21] Florin A, Lambert C, Sanchez C, Zappia J, Durieux N, Tieppo AM (2020). The secretome of skeletal muscle cells: a systematic review. Osteoarthr Cartil Open.

[22] Biolo G, Cederholm T, Muscaritoli M (2014). Muscle contractile and metabolic dysfunction is a common feature of sarcopenia of aging and chronic diseases: From sarcopenic obesity to cachexia. Clin Nutr.

[23] Poonpet T, Honsawek S (2014). Adipokines: biomarkers for osteoarthritis?. World J Orthop.

[24] Bren-Mattison Y, Hausburg M, Olwin BB (2011). Growth of limb muscle is dependent on skeletal-derived Indian hedgehog. Dev Biol.

[25] Shen H, Grimston S, Civitelli R, Thomopoulos S (2015). Deletion of connexin43 in osteoblasts/osteocytes leads to impaired muscle formation in mice. J Bone Miner Res.

[26] Qu Z, Yang F, Yan Y, Huang J, Zhao J, Hong J (2021). A Mendelian randomization study on the role of serum parathyroid hormone and 25-hydroxyvitamin D in osteoarthritis. Osteoarthr Cartil.

[27] Funck-Brentano T, Nethander M, Movérare-Skrtic S, Richette P, Ohlsson C (2019). Causal factors for knee, hip, and hand osteoarthritis: a mendelian randomization study in the UK biobank. Arthritis Rheumatol.

[28] Lyu L, Cai Y, Xiao M, Liang J, Zhang G, Jing Z (2022). Causal relationships of general and abdominal adiposity on osteoarthritis: a two-sample mendelian randomization study. J Clin Med.

[29] McCarthy MI, Abecasis GR, Cardon LR, Goldstein DB, Little J, Ioannidis JPA (2008). Genome-wide association studies for complex traits: consensus, uncertainty and challenges. Nat Rev Genet.

[30] Ebrahim S, Smith GD (2008). Mendelian randomization: can genetic epidemiology help redress the failures of observational epidemiology?. Hum Genet.

[31] Skrivankova VW, Richmond RC, Woolf BAR (2021). Strengthening the reporting of observational studies in epidemiology using mendelian randomisation (STROBE-MR): explanation and elaboration. BMJ.

[32] Boer CG, Hatzikotoulas K, Southam L (2021). Deciphering osteoarthritis genetics across 826,690 individuals from 9 populations. Cell.

[33] Pei Y-F, Liu Y-Z, Yang X-L, Zhang H, Feng G-J, Wei X-T (2020). The genetic architecture of appendicular lean mass characterized by association analysis in the UK biobank study. Commun Biol.

[34] Jones G, Trajanoska K, Santanasto AJ, Stringa N, Kuo C-L, Atkins JL (2021). Genome-wide meta-analysis of muscle weakness identifies 15 susceptibility loci in older men and women. Nat Commun.

[35] Holmes MV, Ala-Korpela M, Smith GD (2017). Mendelian randomization in cardiometabolic disease: challenges in evaluating causality. Nat Rev Cardiol.

[36] Bowden J, Smith GD, Haycock PC, Burgess S (2016). Consistent estimation in mendelian randomization with some invalid instruments using a weighted median estimator. Genet Epidemiol.

[37] Verbanck M, Chen C-Y, Neale B, Do R (2018). Detection of widespread horizontal pleiotropy in causal relationships inferred from mendelian randomization between complex traits and diseases. Nat Genet.

[38] Judd DL, Thomas AC, Dayton MR, Stevens-Lapsley JE (2014). Strength and functional deficits in individuals with hip osteoarthritis compared to healthy, older adults. Disabil Rehabil.

[39] Karlsson MK, Magnusson H, Cöster MC, vonSchewelov T, Karlsson C, Rosengren BE (2014). Patients with hip osteoarthritis have a phenotype with high bone mass and low lean body mass. Clin Orthop Relat R.

[40] Andrews JS, Gold LS, Nevitt M, Heagerty PJ, Cawthon PM (2021). Appendicular lean mass, grip strength, and the development of knee osteoarthritis and knee pain among older adults. Acr Open Rheumatol.

[41] Misra D, Fielding RA, Felson DT, Niu J, Brown C, Nevitt M (2019). Risk of knee osteoarthritis with obesity, sarcopenic obesity, and sarcopenia. Arthritis Rheumatol.

[42] Lovett M, Negm A, Ioannidis G, Petrucelli D, Winemaker M, Adachi JD (2021). Identifying patients with osteoarthritis at risk of sarcopenia using the SARC-F. Can Geriatrics J.

[43] Cruz-Jentoft AJ, Bahat G, Bauer J, Boirie Y, Bruyère O, Cederholm T (2018). Sarcopenia: revised European consensus on definition and diagnosis. Age Ageing.

[44] Cruz-Jentoft AJ, Baeyens JP, Bauer JM, Boirie Y, Cederholm T, Landi F (2010). Sarcopenia: European consensus on definition and diagnosis: report of the European working group on sarcopenia in older People. Age Ageing.

[45] Studenski SA, Peters KW, Alley DE, Cawthon PM, McLean RR, Harris TB (2014). The FNIH sarcopenia project: rationale, study description, conference recommendations, and final estimates. J Gerontol Ser.

[46] Chaisson CE, Zhang Y, Sharma L, Kannel W, Felson DT (1999). Grip strength and the risk of developing radiographic hand osteoarthritis: results from the framingham study. Arthritis Rheum.

[47] Sharma L, Dunlop DD, Cahue S, Song J, Hayes KW (2003). Quadriceps strength and osteoarthritis progression in malaligned and lax knees. Ann Intern Med.

[48] Giudice J, Taylor JM (2017). Muscle as a paracrine and endocrine organ. Curr Opin Pharmacol.

[49] Veronese N, Punzi L, Sieber C, Bauer J, Reginster J-Y, Society O behalf of the TFG on “Arthritis” of the EGM (2018). Sarcopenic osteoarthritis: a new entity in geriatric medicine?. Eur Geriatr Med.

[50] Pickering M-E, Chapurlat R (2020). Where two common conditions of aging meet: osteoarthritis and sarcopenia. Calcif Tissue Int.

[51] Stenholm S, Harris TB, Rantanen T, Visser M, Kritchevsky SB, Ferrucci L (2008). Sarcopenic obesity: definition, cause and consequences. Curr Opin Clin Nutr.

[52] Berenbaum F, Eymard F, Houard X (2013). Osteoarthritis, inflammation and obesity. Curr Opin Rheumatol.

[53] Liu C, Liu N, Xia Y, Zhao Z, Xiao T, Li H (2022). Osteoporosis and sarcopenia-related traits: a bi-directional mendelian randomization study. Front Endocrinol.

[54] Pegreffi F, Balestra A, Lucia OD, Smith L, Barbagallo M, Veronese N (2023). Prevalence of sarcopenia in knee osteoarthritis: a systematic review and meta-analysis. J Clin Med.

